# Phylogenetic Relations and High-Altitude Adaptation in Wild Boar (*Sus scrofa*), Identified Using Genome-Wide Data

**DOI:** 10.3390/ani14202984

**Published:** 2024-10-16

**Authors:** Shiyong Fang, Haoyuan Zhang, Haoyuan Long, Dongjie Zhang, Hongyue Chen, Xiuqin Yang, Hongmei Pan, Xiao Pan, Di Liu, Guangxin E

**Affiliations:** 1 College of Animal Science and Technology, Southwest University, Chongqing 400716, China; fsy2022swu@163.com (S.F.); swuzhanghy@163.com (H.Z.); asrimoom@gmail.com (H.L.); 2 Institute of Animal Husbandry, Heilongjiang Academy of Agricultural Sciences, Harbin 150086, China; djzhang8109@163.com; 3 Chongqing Animal Husbandry Technology Extension Station, Chongqing 401121, China; hy106@163.com; 4 College of Animal Science and Technology, Northeast Agricultural University, Harbin 150030, China; xiuqinyang@neau.edu.cn; 5 Chongqing Academy of Animal Sciences, Chongqing 408599, China; panhm_2118@163.com; 6 Chongqing Hechuan Animal Husbandry Station, Chongqing 401520, China; cqshcqnjj163@163.com

**Keywords:** wild boar, Tibetan, phylogenetic, wide genome, altitude adaptation

## Abstract

**Simple Summary:**

The genetic diversity and phylogenetic relationships among wild boars in major regions of the world were assessed using genome-wide data from the Qinghai–Tibet Plateau (QTP), southern and northern regions of China, Europe, Northeast Asia, and Southeast Asia. We clarified the close phylogenetic relationship of QTP wild boars with those in southern China. In addition, genome-wide selection signal analysis based on cross-population extended haplotype homozygosity, fixation index parameters, and run of homozygosity island recognition technology was performed to obtain a series of candidate genes related to the plateau adaptability of wild boar, such as *TSC2*, *TELO2*, *VCP*, *SLC5A1*, and *SLC5A4*. In particular, *SLC5A1* and *SLC5A4* are involved in glucose homeostasis and may be the key to the plateau adaptability of wild boars. This study enhances our understanding of the genetic mechanism of high-altitude adaptation in wild boars.

**Abstract:**

The Qinghai–Tibet Plateau (QTP) wild boar is an excellent model for investigating high-altitude adaptation. In this study, we analyzed genome-wide data from 93 wild boars compiled from various studies worldwide, including the QTP, southern and northern regions of China, Europe, Northeast Asia, and Southeast Asia, to explore their phylogenetic patterns and high-altitude adaptation based on genome-wide selection signal analysis and run of homozygosity (ROH) estimation. The findings demonstrate the alignment between the phylogenetic associations among wild boars and their geographical location. An ADMIXTURE analysis indicated a relatively close genetic relationship between QTP and southern Chinese wild boars. Analyses of the fixation index and cross-population extended haplotype homozygosity between populations revealed 295 candidate genes (CDGs) associated with high-altitude adaptation, such as *TSC2*, *TELO2*, *SLC5A1*, and *SLC5A4*. These CDGs were significantly overrepresented in pathways such as the mammalian target of rapamycin signaling and Fanconi anemia pathways. In addition, 39 ROH islands and numerous selective CDGs (e.g., *SLC5A1*, *SLC5A4*, and *VCP*), which are implicated in glucose metabolism and mitochondrial function, were discovered in QTP wild boars. This study not only assessed the phylogenetic history of QTP wild boars but also advanced our comprehension of the genetic mechanisms underlying the adaptation of wild boars to high altitudes.

## 1. Introduction

The wild boar (*Sus scrofa*) represents one of the most widely distributed terrestrial mammals; they have been extensively distributed throughout the Eurasian continent for the past one million years [1]. Approximately 3–4 million years ago, wild boars emerged as a new species in the tropical forests of Southeast Asia (SEA) and then spread to the Asian continent in the middle of the Pleistocene [2]. The Qinghai–Tibet Plateau (QTP) wild boar has long survived the harsh environment of the Tibetan Plateau, one of the highest and largest plateaus in the world. They are mainly distributed in mountain grasslands, low meadows, and river valleys at an altitude of about 2500–4000 m [3,4]. Current research has advanced scientific hypotheses regarding the historical migration paths of wild boars in East Asia [1]; the evolution and phylogeny of wild boars in Tibet have also been reported [3,5,6]. However, the migration history of QTP wild boars on the QTP is not entirely known.

As one of the most widely distributed terrestrial mammals, wild boars possess excellent environmental adaptability and disease resistance, which make them important model animals for the study of environmental adaptation [1,7,8]. Through comparative analyses of the genomes of QTP wild boars and domestic pigs, a series of CDGs has been identified as being related to the adaptability of QTP wild boars to the environment of the region; these CDGs include genes involved in the response to hypoxia (*HIF1A*, *PIK3C2G*, and *DPP4*), DNA repair (*BCL3*, *REV1*, and *USF1*), and energy metabolism (*ADAMTS9*, *CPEB4*, and *DGAT1*) [3,9]. In addition. the Tibetan pig population in the QTP region serves as a source of unique CDGs for adaptation to the harsh environment of the plateau [10,11,12].

However, due to the divergence in natural and artificial selection directions experienced by wild boars and domestic pigs over a long period of time, they exhibit significant differences in their physiological phenotypes and genetic backgrounds [13,14]. Therefore, analyzing the population genetic structure and genomic differences among QTP wild boars or other lowland wild boars can help better identify the genetic basis of high-altitude adaptation in the same subspecies of animals. In this study, a massive volume of published whole-genome data on Eurasian wild boars was utilized to explore their phylogenetic patterns and high-altitude adaptation based on genome-wide selection signal analysis, which may improve our understanding of the genetic basis of gene flow migration and high-altitude adaptation among wild boars.

## 2. Materials and Methods

### 2.1. Sample Information and Data Quality Control

This study utilized 93 public genomes of wild boar species obtained from various sources worldwide [3,5,15,16,17]. These genomes comprised 28 samples from the QTP, 16 from southern China (SCN), 5 from northern China (NCN), 12 from Northeast Asia (NEA), 30 from Europe (EU), and 2 from SEA (Table S1). The SNP genotype datasets of all individuals were downloaded from the Genome Variation Map (https://ngdc.cncb.ac.cn/gvm/, accessed on 16 August 2022) and aligned to the pig reference genome (Sscrofa 10.2) (https://www.ncbi.nlm.nih.gov/datasets/genome/GCF_000003025.5/, accessed on 19 August 2022).

A total of 93 samples were extracted using BCFtools, which resulted in a dataset of 87,149,692 autosomal single-nucleotide polymorphisms (SNPs) out of a total of 90,901,469 SNPs. Subsequently, 18,270,383 SNPs were retained after filtration using PLINK software (PLINK v1.90b6.24 64 bit [6 June 2021]; https://www.coggenomics.org/link/1.9/, accessed on 13 September 2022) [18] with a minimum allele frequency (MAF) of ≥0.05 and a call rate of ≥0.9 for the genome-wide selection signal analysis (GWSA) of the high-altitude adaptation of wild boars. In addition, the “indep pairwise” command was set to 50 10 0.1 in Plink V1.9 software for filtering, and 559,949 SNPs were obtained for the subsequent population phylogenetic analysis and diversity estimation.

### 2.2. Phylogenetic Relation and Population Genetic Analysis

The genetic distances between all individuals were calculated using VCF2Dis (https://github.com/hewm2008/VCF2Dis, accessed on 2 October 2022). The maximum-likelihood phylogenetic network was constructed using FastME 2.1.6.2 [19] and visualized using iTOL (https://itol.embl.de/, accessed on 21 April 2023). A principal component analysis (PCA) was performed, and visualization of the results was completed using the R program (http://www.r-project.org/, accessed on 5 October 2022). In addition, the pairwise difference (F_ST_) between populations was calculated using SMARTPCA (https://www.hsph.harvard.edu/alkes-price/software/, accessed on 14 April 2024), and the neighbor-net graph was visualized using Splitstree CE (V6.3.0-beta, built 19 Mar 2024) [20]. ADMIXTURE 1.3.0 software [21] was used to estimate the phylogenetic clustering and population structure. When *K* = 2–10, the number of repeats was 20, and the minimum cross-validation (CV) error was used to determine the most reliable *K* value. The linkage disequilibrium (LD) of each population was calculated using PopLDdecay (V3.40) software [22], and the findings of the demographic history inference analysis of each population with typical samples were visualized using SMCpp (V1.15.2) software [23].

### 2.3. Run of Homozygosity (ROH) Detection and Analysis

The ROHs were analyzed using PLINK software (PLINK v1.90b6.21 64-bit [19 October 2020]). The data quality control criteria were as follows: SNP detection rate ≥ 95%, MAF ≥ 0.05, and SNPs with a *p*-value of ≥10-6 in the Hardy–Weinberg equilibrium. A total of 14,299,949 out of 87,149,692 SNPs were retained for subsequent analysis.

The density distribution of SNPs on each chromosome was plotted and viewed using plot snp_densiny0 in the R package HandyCNV (v1.1.6) (https://github.com/JH-Zhou/HandyCNV, accessed on 23 January 2024). The detection method involved the use of a sliding window approach with a window size of 50 SNPs. Up to two genotypes were allowed to be missing in each sliding window, and one possible heterozygous genotype was permitted in the ROH. The maximum gap between SNPs composing an ROH was set to 100 kb, and the minimum density of SNPs forming the ROH was set to 50 kb. In addition, the minimum length of ROH fragments was set to 500 kb, and the minimum number of SNPs required to constitute an ROH was set to 100 based on its physical length. The computed ROHs were categorized into four length groups: <1, 1–2, 2–4, and 8–16 Mb. Furthermore, various metrics were calculated for each population: the total number, total length, average number, and average length of ROHs and the number of ROH islands. The inbreeding coefficient (FROH) was calculated following the method described by McQuillan et al. (2008). This step involved dividing the sum of lengths of ROH fragments on autosomes by the sum of the total length of autosomes [24]. The results of the ROH analysis were visualized using Rstudio (https://www.r-project.org/?ref=vijayraghunathan.ghost.io, accessed on 27 January 2024). ROH islands were determined using a threshold of the window with a 25% sample size or more within the population and identified based on the SNP distribution exceeding this threshold in the genome.

### 2.4. Genome-Wide Selection Signal Analysis Based on F_ST_ and XP-EHH

The GWSA was performed on QTP wild boars in comparison with non-QTP wild boars using the F_ST_ and XP-EHH. The F_ST_ was calculated using vcftools (v0.1.16) [25], with the window and step length set to 40 and 20 kb, respectively. The XP-EHH was calculated using selscan (v2.0.0), with a 40 kb size for each window [26]. Positive XP-EHH scores represented the selective signals that occur in QTP wild boar. CDGs included the intersecting genes of the top 5% of windows from both parameters (F_ST_ and XP-EHH). Gene annotation was performed using ANNOVAR (ANNOate VARiation, https://annovar.openbioinformatics.org/en/latest/, accessed on 20 April 2024) software based on the annotation file of the ENSEMBL database (http://useast.ensembl.org, accessed on 20 April 2024). A functional enrichment analysis of the CDGs was conducted using the KOBAS database (http://bioinfo.org/kobas/, accessed on 5 May 2024) for Gene Ontology (GO) and Kyoto Encyclopedia of Genes and Genomes (KEGG) analyses. The significance threshold for enrichment was defined as *p* < 0.05.

## 3. Results

**Figure 1:**
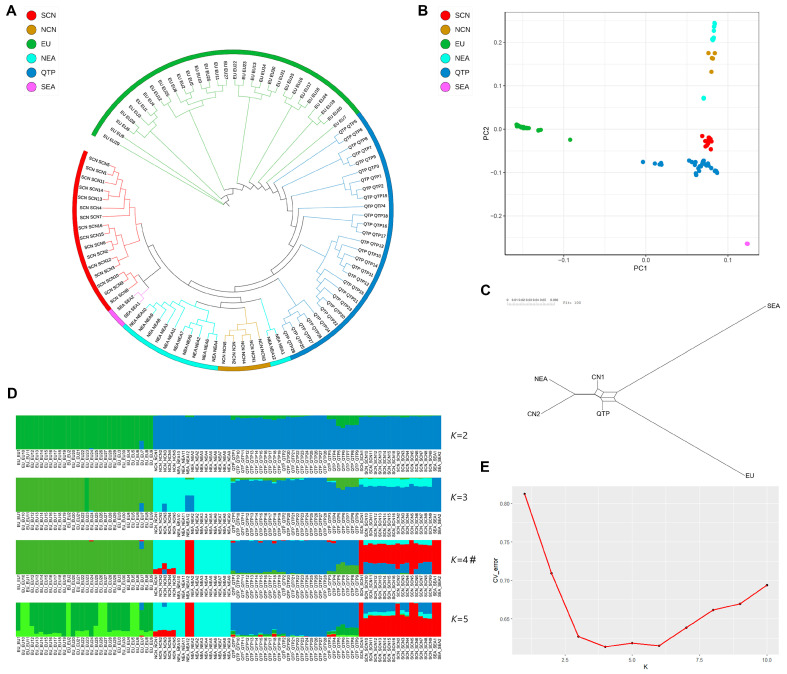
Phylogenetic analysis and population structure of worldwide wild boars. (**A**) Genome-wide phylogenetic trees of wild boar populations. Each color represents the wild boar population in a different region, including the SCN wild boar population (SCN), the NCN wild boar population (NCN), the EU wild boar population (EU), the NEA wild boar population (NEA), the QTP wild boar population (QTP), and the SEA wild boar population (SEA). (**B**) Principal component analysis, based on all available data, divided into six groups by region. (**C**) Neighbor-net graph of worldwide wild boar populations using the pairwise difference (Fs_T_). (**D**) Analysis of the population structure of each wild boar population. The *K* value is the number of assumed ancestral populations, which was 2 to 5. #: The most reliable *K* value was 4, which had the minimum CV error. (**E**) Cross-validation error for each *K* value (*K* = 1–10).

An initial comprehensive study was conducted on the phylogenetic associations and genetic structure of wild boar populations across various geographical regions. The resulting phylogenetic network of wild boars (Figure 1A) revealed the grouping of the populations in EU and Asia into distinct branches. Conversely, the wild boar populations in NEA and NCN exhibited similar phylogenetic associations, as did the populations in SEA and SCN. Thus, the phylogenetic associations among wild boar populations in Eurasia, including those on the QTP, corresponded to their geographical distribution. The PCA results (Figure 1B) demonstrated a significant separation between the EU and Asian populations, with the QTP wild boars exhibiting the closest population structure to that of SCN wild boars. Furthermore, the result of the neighbor-net graphs revealed that European and Asian wild boars formed two separate branches, and the smallest divergence (Fs_T_ = 0.069) was observed between QTP and SCN wild boars (Figure 1C). The findings of an ADMIXTURE analysis (Figure 1D) indicated that when *K* = 2, the EU and Asian wild boars belonged to two distinct lineages, whereas the QTP wild boars were under the Asian wild boar cluster. Moreover, when the optimal *K* value of 4 was considered, the Asian wild boar population further segregated into two lineages: NEA (which includes NCN wild boars) and SCN. Notably, the population structure of the QTP wild boars closely resembles that of the SCN wild boars.

**Figure 2:**
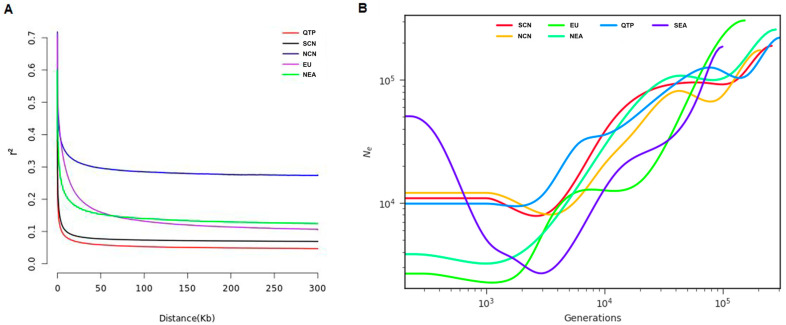
Population LD decay and demographic history inference analysis of wild boar populations. (**A**) LD decay of wild boar populations, including the southern Chinese wild boar population (SCN), northern Chinese wild boar population (NCN), European wild boar population (EU), Northeast Asian wild boar population (NEA), and Qinghai–Tibet Plateau wild boar population (QTP). (**B**) Effective population sizes of different wild boar populations, inferred from autosomes.

The LD analysis results revealed faster LD decay for QTP wild boars than for other geographical populations, with a similar pattern observed in the SCN wild boars but at a faster rate than that among the NCN wild boars (Figure 2A). An examination of the effective population size (*Ne*) offered insights into its decline over time. Remarkably, the worldwide wild boar population started exhibiting a significant decrease in its *Ne* approximately 100,000 years ago (Figure 2B). The trend of *Ne* among wild boars in the SCN network mirrored that among NCN wild boars. Intriguingly, the *Ne* of the QTP wild boars slowly declined over one million years, with a substantial acceleration occurring approximately 6000 years ago. Ultimately, the QTP wild boars had an *Ne* comparable to that of wild boars in SCN.

**Figure 3:**
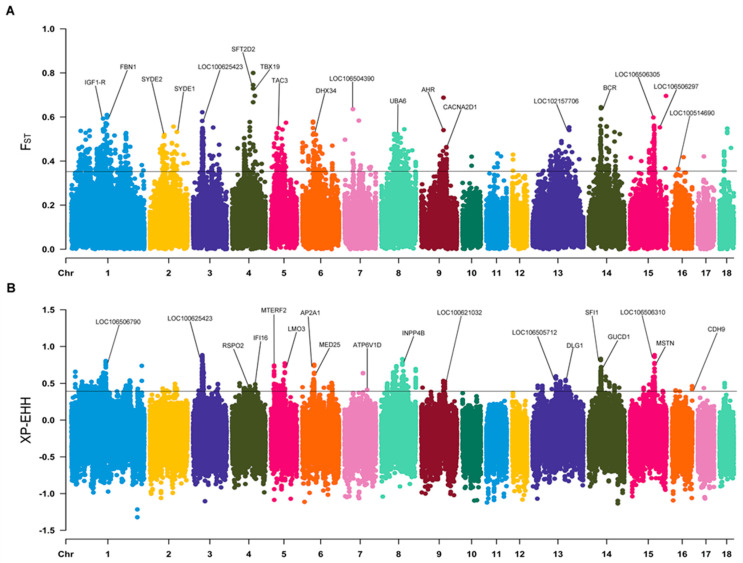
Genome-wide selective signal analysis of worldwide wild boars to identify the high-altitude adaptability-related genes in Qinghai–Tibet Plateau wild boars. (**A**) Manhattan map of F_ST_ between groups. (**B**) Manhattan map of XP-EHH between groups.

This study also presents the results of a GWSA of 93 wild boar genomes, which were used to explore the genetic basis of high-altitude adaptation in QTP wild boars. Totals of 2058 and 354 CDGs were obtained using the F_ST_ and XP-EHH (top 5%; F_ST_ ≥ 0.34 and XP-EHH ≥ 0.39) (Figure 3), respectively. Furthermore, 295 interacting CDGs (e.g., *TSC2*, *TELO2*, and *SLC5A1*) were identified via both methods (Table S2). A functional enrichment analysis of these CDGs revealed the significant enrichment of 426 GO items (Table S3) and 200 KEGG pathways (Table S4) (*p* < 0.05). Importantly, these genes are widely enriched in physiological functions related to altitude adaptation. Such functions include autophagy (e.g., *TSC2*, *WDR24*, and *NPRL3*), DNA damage response and repair (e.g., *TELO2*, *REV3L*, and *SLX4*), and glucose homeostasis (*SLC5A1* and *SLC5A4*).

The ROH analysis showed 22,726 continuous homozygous regions detected in all the samples, with an average of 252.5 ROHs per individual. Furthermore, 2457 continuous homozygous regions were detected in the QTP wild boar samples. The average ROH number for each sample was approximately 87.8 (Table 1). SNPs on the autosomes had an average density of 5820 SNPs/Mb, of which 371 regions had an SNP density less than 3052 SNPs/Mb, and 431 regions had a density greater than 5820 SNPs/Mb (Figure S1).

In addition, the ROH lengths were mostly less than 8 Mb, and only European wild boar populations exhibited ROH lengths greater than 8 Mb. The number of ROHs decreased with an increase in their length, with ROHs shorter than 1 Mb being the most frequently observed (70.9% of the total) and averaging approximately 179 per sample. Regions with a length of less than 1 Mb accounted for the greatest proportion (84.5%) among all wild boars, with an average of approximately 74.1 ROHs per sample. The NEA wild boar population exhibited the greatest number of ROH islands (945) (Table S5) and the greatest average number of individual ROHs (457.42) (Table S6). The SCN wild boar population had the lowest number of ROH islands (27), whereas the NCN wild boar population had the lowest average number of individual ROHs (76.8). The NEA wild boar population had the highest estimated FROH (0.172), whereas the NCN wild boars had the lowest (0.023) (Table S7).

**Figure 4:**
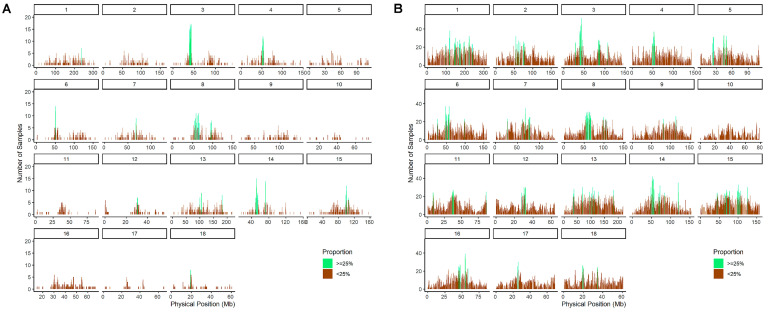
ROH proportions among the populations. The width of the bar chart in the figure is 20KB, and green marks the regions of the genome with ROH population frequencies greater than 25%. (**A**) ROH proportions in the Qinghai–Tibet Plateau wild boar population. (**B**) ROH proportions in wild boar populations not distributed on the Qinghai–Tibet Plateau, including the northern Chinese wild boar population, southern Chinese wild boar population, European wild boar population, and Northeast Asian wild boar population.

Among the QTP wild boars, 39 ROH islands (sample size ≥ 25%) were identified in other wild boar populations across 12 chromosomes, with a cumulative length of approximately 29.08 Mbp. The ROH islands were primarily located on chromosomes 1, 8, 13, 14, and 15, with the highest number of regions observed on chromosome 8 (nine regions) (Figure 4A). Conversely, in other wild boar populations, 1895 ROH islands (sample size ≥ 25%) were found across 18 chromosomes, with a cumulative length of approximately 1772.88 Mbp. These additional ROH islands were mainly located on chromosomes 1, 2, 13, 14, and 15 (Figure 4B).

Furthermore, 74 CDGs were annotated from 18 ROH islands found in the QTP wild boar (Table S8), of which 15 CDGs were located in ROH islands and were revealed by the F_ST_ and XP-EHH (Table S9). The GO functional enrichment analysis revealed the enrichment of 51 out of 74 CDGs in 300 GO terms, 124 of which were significantly enriched (Table S10). These significantly enriched terms included GO terms related to mitochondrial homeostasis, such as positive regulation of mitochondrial membrane potential (*VCP*), Vcp-npl4-ufd1 AAA ATPase complex (*VCP*), and ATP metabolic process (*VCP*). In addition, some GO terms were related to glucose homeostasis, such as glucose–sodium symporter activity (*SLC5A1* and *SLC5A4*) and glucose transmembrane transport (*SLC5A1* and *SLC5A4*). The KEGG pathway annotation analysis revealed the enrichment of 23 out of 74 CDGs in 103 KEGG signaling pathways, with 30 pathways displaying significant enrichment (Table S11). These pathways included those related to glucose metabolism, such as carbohydrate digestion and absorption (*SLC5A1* and *HK1*) and insulin secretion (*CHRM3*, *KCNN4*, and *CREB3*). Notably, these CDGs were significantly enriched in GO and KEGG terms associated with high-altitude adaptation, such as glucose homeostasis, mitochondrial homeostasis, and protein quality control.

## 4. Discussion

The migration route of the global wild boar population can be traced back to the Mekong River region in SEA, which has been confirmed as the primary source of the wild ancestors of domestic pigs in East Asia [14]. The phylogenetic results of this study revealed significant genetic differences between EU and Asian wild boars [27]. In addition, the ADMIXTURE analysis unveiled the similar genetic backgrounds of Tibetan and Asian wild boars when *K* = 2. Specifically, they are closely related to wild boars from SCN when *K* = 4 and distantly related to those from NEA, which includes Korea, Japan, and NCN.

The geographical distribution and genetic background of terrestrial wildlife populations are heavily influenced by past climatic and geological changes [28,29]. The cold climate during the last glacial period caused population bottlenecks among wild boars since the late Quaternary [30]. The results of the population history analysis indicate that the *Ne* of EU wild boars exhibited a significant decrease approximately 15,000–100,000 years ago. However, the *Ne* of Chinese wild boars declined at a slower rate during this period, and SCN wild boars showed a lower decrease in their *Ne* than did NCN wild boars, consistent with the assumption that climate change during the ice age had a smaller effect on SCN wild boars than on NCN wild boars [31]. Furthermore, wild boars from the QTP showed similar rates of LD decay and *Ne* in the past 3000 years to those in SCN wild boars, which indicates that this wild boar population had a greater genetic diversity than the wild boars in NEA and EU and a similar diversity to SCN wild boars. These results may be attributed to the fact that the lower elevations of the QTP were not covered by ice sheets during the last glacial period, which provided an important glacial refuge for animals [32]. Moreover, the *Ne* of QTP wild boars began to decline significantly again 6000 years ago, and this phenomenon may be related to large-scale human migration and settlement on the QTP [33]. Choi et al. proposed three primary migration routes of wild boars (clades C1–C3) on the Eurasian continent [1]. Their findings indicated that Chinese wild boars followed the third migration route (clade C3). This outcome suggests that the ancestors of QTP wild boars entered China and gradually dispersed to the QTP from SCN. However, the QTP is surrounded by towering mountains, and only its northeastern edge gradually descends to lower altitudes, according to Yang et al. [10]. This particular situation challenges the hypothesis that wild boars migrated from the lowland regions of SCN to the QTP. Interestingly, mtDNA D-loop data indicate that the East Asian boar lineage originating from the Mekong River may have progressively spread through Sichuan, Chongqing, and northern Yunnan into Gansu and the eastern Qinghai region and potentially reached the northeastern QTP [34]. Moreover, evidence suggests the migration of Tibetan sheep to the northeastern part of the QTP through Shanxi and Gansu; in addition, prehistoric humans gradually expanded their presence from the Loess Plateau to the northeastern QTP and ultimately settled there [33,35]. Hence, the present study suggests that the ancestors of QTP wild boars gradually migrated from southwest China and spread to the northwest Loess Plateau, then to the northeastern part of the QTP, and finally to the QTP itself.

Compared with animals residing in flat areas, those inhabiting the QTP face distinct challenges, such as low oxygen pressure and intense ultraviolet (UV) radiation. Consequently, species dwelling on plateaus must coevolve with their ecological environment in terms of their physiology and heredity over extended periods to ensure the persistence of their populations [36]. In this study, GWSA revealed a comprehensive set of CDGs associated with high-altitude adaptation, including *TSC2*, *WDR24*, *TELO2*, and *SLC5A1*. Notably, a considerable portion of these genes is associated with hypoxia, autophagy, the DNA damage response, and glucose metabolism.

The plateau area features a low oxygen concentration, which poses considerable ecological challenges. Candidate genes involved in the hypoxic response are commonly detected in species living in the QTP due to the high-altitude and low-oxygen environment of the QTP region [37,38,39]. The *RXFP1* gene participates in the lung development of Tibetan pigs and can protect astrocytes from hypoxia as a receptor of the relaxin family, which contributes to hypoxia adaptation in Tibetan pigs [40,41,42]. A previous study on the species of *Bos* introgression revealed that the hypoxia-inducible factor 3a (*HIF3a*) gene involved in the hypoxia response pathway from yaks has infiltrated Tibetan cattle, which have survived for a long time on the Tibetan Plateau. As an important member of the HIF family, HIF3-α plays a key role in the hypoxic transcription response. HIF-3α protein can act as an oxygen-regulated transcriptional activator in response to hypoxia, which may aid Tibetan cattle in adapting to the high-altitude and low-oxygen environment of the Tibetan Plateau [43,44]. A comparative analysis of Tibetan partridges with nine other lowland bird species revealed a positive selection signal for the *CREBBP* gene, which is an important component of the HIF-1 signaling pathway involved in the cellular response to hypoxia and its regulation [45]. Creb-binding protein, the product of *CREBBP*, can bind to HIF-2α as a transcriptional coactivator; a reduction in its transcriptional activity leads to a decrease in HIF-2α activity [46].

Animal cells experience metabolic disorders and oxidative stress damage in low-oxygen environments. Autophagy plays a critical role in the restoration of the body’s metabolic balance through the degradation of damaged cells and their components. This process serves as a crucial regulatory mechanism underlying adaption and adjustment to low-oxygen environments. Notably, autophagy effectively reduces the production and accumulation of reactive oxygen species [47,48].

In this study, a series of genes involved in mediating autophagy under hypoxic conditions was identified, with *WDR24* and *TSC2* playing crucial roles. The mammalian target of rapamycin complex 1 (mTORC1) is an essential component of the mTOR pathway, which is crucial for mediating autophagy under environmental stress [49]. Under hypoxia and energy-limited conditions, *WDR24* can coordinate mTORC1 and lysosomal activities by influencing the GTPase-activating protein towards Rags complex (GATOR) 2 complex, which affects cell homeostasis [50]. *TSC2*, on the other hand, is a target of mTORC1 inhibition. This gene indirectly modulates mTORC1 activity and influences autophagy through the phosphorylation of protein kinase B and extracellular response kinase [51]. In addition, AMP-activated protein kinase activates *TSC2* to inhibit the effects of mTORC1 and reduce reactive oxygen species production under hypoxic conditions, which alleviates cellular stress [52].

In addition, *NPRL3* and *DEPDC5* play important roles in the regulation of mTORC1 and autophagy. *NPRL3* and *DEPDC5* are key components of the GATOR1 complex, which can mediate autophagy and homeostasis by participating in the regulation of mTORC1 [53]. The physiological function of GATOR1 is regulated by the body’s amino acid level. GATOR1 participates in mediating autophagy activation and inhibits mTORC1 to reduce amino acid consumption and maintain cell homeostasis under the restriction of the amino acid level [54]. Conversely, GATOR1 deficiency results in the response failure of the mTORC1 signaling pathway to nutritional deficiencies in the body [55].

Furthermore, the animals inhabiting the QTP face a constant threat to their genomic integrity due to the damaging effects of strong solar UV radiation [56,57]. Notably, this study identified the *REV3L*, *TELO2*, and *SLX4* genes as key players in this process. *REV3L*, the catalytic subunit of DNA polymerase zeta, is a key component involved in translesion DNA synthesis across damaged DNA and can effectively provide cell protection against various types of endogenous or exogenous DNA damage [58]. *REV3L* is a key component of the DNA replication fork protection mechanism; it can effectively prevent the genomic instability caused by DNA replication stress and enables cells to resist DNA damage induced by various sources, such as UV light [59]. *TELO2* interacts with members of the phosphoinositide 3-kinase family, such as ataxia telangiectasia mutations, ataxia telangiectasia, and Rad3-associated mutations, and contributes to the regulation of the DNA damage response. This gene also coordinates the cellular response to replication stress, which ensures the preservation of genomic integrity [60,61]. *SLX4* can act as a scaffolding protein for DNA repair by coordinating structure-specific endonucleases and participate in a variety of DNA repair pathways [62,63]. Furthermore, *TELO2* and *SLX4* are enriched in the Fanconi anemia pathway, which is primarily responsible for the repair of DNA strand cross-links, maintenance of genome stability, stabilization of replication forks, and regulation of cell division [64]. Consequently, the *REV3L*, *TELO2*, and *SLX4* genes possibly take part in conferring resistance to intense UV radiation in wild boars dwelling in high-altitude plateau environments.

Mitochondria and their maintenance of homeostasis contribute to adaption to low-oxygen plateau environments [65]. Mitochondria-associated degradation repairs or eliminates damaged mitochondria and protects them from oxidative stress [65,66]. This study identified a series of CDGs related to mitochondrial homeostasis, such as *VCP*, through ROH analysis, along with a key enzyme in mitochondrial outer membrane protein degradation [67] that participates in the extraction of ubiquitinated mitochondrial outer membrane proteins and their presentation to the proteasome for degradation. This process is essential for the maintenance of mitochondrial outer-membrane protein homeostasis and mitochondrial health [68]. *VCP* mutations lead to severe structural and functional damage to mitochondria, which results in mitochondrial degeneration, motor neuron degeneration, and increased autophagy in mice [69].

Furthermore, in high-altitude environments, energy metabolism in animals faces important challenges, namely, hypoxia and hypothermia. Therefore, glucose homeostasis must be maintained for survival in alpine and low-oxygen plateau regions [70,71]. This study identified a series of CDGs related to glucose homeostasis, such as *SLC5A1* and *SLC5A4*, through ROH, F_ST_, and XP-EHH analyses. SGLT1 contributes to the rapid absorption of glucose and galactose in the intestine and regulates glycolysis [72,73]. Previous evidence suggests that SGLT1 inhibition can improve glucose homeostasis through reduced glucose absorption in the gut and the increased release of glucagon-like peptide-1 [74]. Impaired glucose utilization due to intestinal tight junction injury has been associated with the decreased expression of *SLC5A1* [75]. SGLT1 can prevent the degeneration of brain tissue neurons, which protects animals from brain damage in high-altitude environments with low oxygen levels and cold temperatures [76,77]. In addition, SGLT1 may increase ATP concentrations in myocardial cells during myocardial ischemia or hypoglycemia, which alleviates myocardial damage caused by oxidative stress, fibrosis, and ischemia–reperfusion [74,76]. SGLT3, which is encoded by *SLC5A4*, also performs an important function in glucose metabolism and homeostasis maintenance. Previous findings have shown that, different from SGLT1, SGLT3 physiologically functions more as a glucose sensor, and its activation can promote glucose transport in SGLT1 [78]. Specifically, SGLT3 is involved in mediating the secretion of incretin hormone glucagon-like peptide-1 and thereby affects glucose metabolism [79].

## 5. Conclusions

This study elucidated the genetic diversity and phylogenetic relations among wild boars in major global regions. Furthermore, a substantial number of CDGs associated with altitude adaptation were identified. These CDGs contribute considerably to our understanding of the genetic mechanisms underlying altitude adaptation among wild boars.

## Figures and Tables

**Table 1:** Mean and standard deviation of ROHs and ROH-based inbreeding (FROH) among global wild boar populations.

Groups	Populations	Samples	Number of ROHs	Number of ROH Islands	Average Length of ROH/Mb	Number of ROHs in Each Sample		F_ROH_	
						Mean ± SD	Interval	Mean ± SD	Interval
Case	QTP	28	2457	39	0.77	87.75 ± 56.73	31.02–144.48	0.025 ± 0.017	0.001–0.064
Control	SCN	16	1258	27	1.03	78.63 ± 66.25	12.38–144.89	0.030 ± 0.034	0.003–0.131
	SCN	5	379	123	0.8	76.8 ± 66.26	10.54–143.06	0.023 ± 0.023	0.001–0.053
	NEA	12	5489	945	1	457.42 ± 238.51	218.92–695.92	0.1720 ± 0.090	0.060–0.372
	EU	30	13,137	800	0.99	453.20 ± 160.22	292.98–613.22	0.1679 ± 0.074	0.025–0.289

## Data Availability

The original data presented in this study are included in the article/Supplementary Materials. Further information is available in the public database Genome Variation Map (https://ngdc.cncb.ac.cn/gvm/).

## Supplementary Materials

The following supporting information can be downloaded at: https://www.mdpi.com/article/10.3390/ani14202984/s1, Figure S1: SNP density distribution on autosomes of Qinghai–Tibet Plateau wild boar. Table S1: Information on sampling of global wild boar populations [3,15,16,17,80]. Table S2: A summary of candidate genes overlapped by F_ST_ and XP-EHH in Qinghai–Tibet Plateau wild boar. Table S3: Gene Ontology (GO) terms enriched based on candidate genes overlapped by F_ST_ and XP-EHH. Table S4: KEGG pathways enriched based on candidate genes overlapped by F_ST_ and XP-EHH. Table S5: Information of ROH islands in global wild boar populations. Table S6: Summary of ROH statistics of global wild boar populations. Table S7: Inbreeding coefficient (FROH) estimation of global wild boar populations based on ROH data. Table S8: Gene functional annotation of ROH island candidate genes in Qinghai–Tibet Plateau wild boars. Table S9: A summary of candidate genes overlapped by ROH, F_ST_, and XP-EHH analyses in Qinghai–Tibet Plateau wild boar. Table S10: Gene Ontology (GO) terms enriched based on annotated genes within ROH islands. Table S11: KEGG pathways enriched based on annotated genes within ROH islands.
