# Supplementary material for: Phylogenetic Relations and High-Altitude Adaptation in Wild Boar (Sus scrofa), Identified Using Genome-Wide Data
Source: Animals (Basel). 2024 Oct 16;14(20):2984. doi: 10.3390/ani14202984 (PMC11503864; doi:10.3390/ani14202984)
Supplement: Supplementary file 1 [file animals-14-02984-s001.zip › Table S7. Inbreeding coefficient (FROH) estimation of global wild boar populations base on ROH data.pdf]

Table S7. Inbreeding coefficient (FROH) estimation of global wild boar populations base on ROH data

Population	Sample	Mean	SD	Min	Max
QTP wild boar	28	0.025385	0.017178	0.000634	0.063847
SCN wild boar	16	0.03043	0.033757	0.003479	0.13146
NCN wild boar	5	0.022888	0.023377	0.000978	0.053397
NEA wild boar	12	0.172032	0.089568	0.059929	0.37295
EU wild boar	29	0.167914	0.073823	0.025391	0.288525
