# Supplementary figures and images for: Phylogenetic Relations and High-Altitude Adaptation in Wild Boar (Sus scrofa), Identified Using Genome-Wide Data
Source: Animals (Basel). 2024 Oct 16;14(20):2984. doi: 10.3390/ani14202984 (PMC11503864; doi:10.3390/ani14202984)

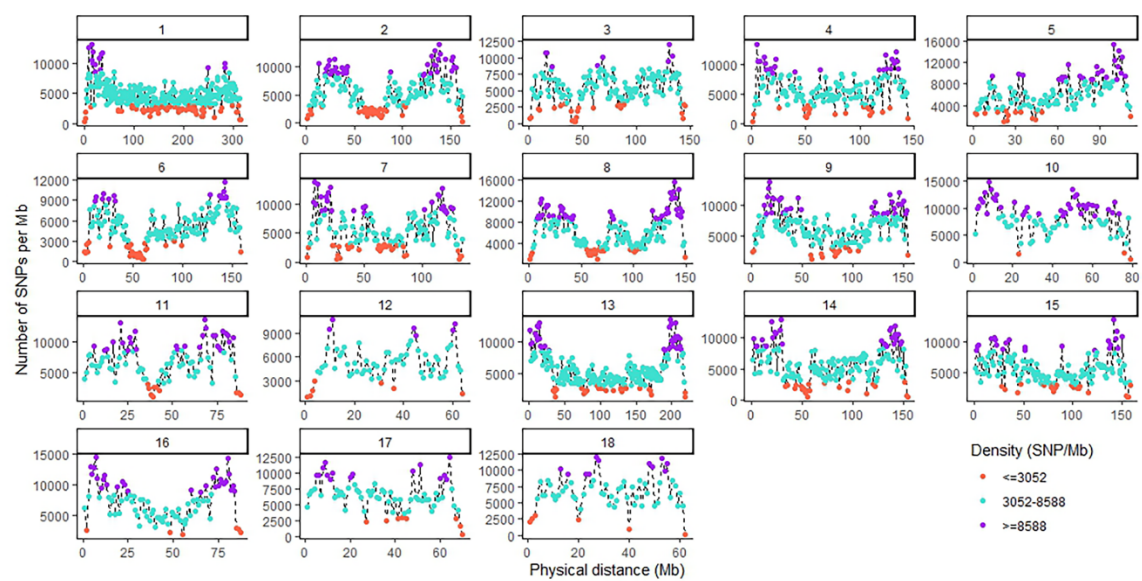

**Figure S1.** SNP density distribution on autosomes of Qinghai-Tibet Plateau wild boar

Supplement: Supplementary file 1 [file animals-14-02984-s001.zip › Figure S1.SNP density distribution on autosomes of Qinghai-Tibet Plateau wild boar.pdf]
